# The role of brain perivascular space burden in early-stage Parkinson’s disease

**DOI:** 10.1038/s41531-021-00155-0

**Published:** 2021-02-05

**Authors:** Ting Shen, Yumei Yue, Shuai Zhao, Juanjuan Xie, Yanxing Chen, Jun Tian, Wen Lv, Chun-Yi Zac Lo, Yi-Cheng Hsu, Tobias Kober, Baorong Zhang, Hsin-Yi Lai

**Affiliations:** 1 Department of Neurology of the Second Affiliated Hospital, Interdisciplinary Institute of Neuroscience and Technology, Key Laboratory of Medical Neurobiology of Zhejiang Province, Zhejiang University School of Medicine, Zhejiang University, Hangzhou, China; 2 Department of Neurology of the Second Affiliated Hospital, Zhejiang University School of Medicine, Zhejiang University, Hangzhou, China; 3 College of Biomedical Engineering and Instrument Science, Key Laboratory for Biomedical Engineering of Ministry of Education, Zhejiang University, Hangzhou, China; 4 Department of Neurology of Sir Run Run Shaw Hospital, Zhejiang University School of Medicine, Zhejiang University, Hangzhou, China; 5 Institute of Science and Technology for Brain-Inspired Intelligence, Fudan University, Shanghai, China; 6 MR collaboration NE Asia, Siemens Healthcare, Shanghai, China; 7 Advanced Clinical Imaging Technology, Siemens Healthcare, Lausanne, Switzerland

**Keywords:** Parkinson's disease, Parkinson's disease

## Abstract

Perivascular space (PVS) is associated with neurodegenerative diseases, while its effect on Parkinson’s disease (PD) remains unclear. We aimed to investigate the clinical and neuroimaging significance of PVS in basal ganglia (BG) and midbrain in early-stage PD. We recruited 40 early-stage PD patients and 41 healthy controls (HCs). Both PVS number and volume were calculated to evaluate PVS burden on 7 T magnetic resonance imaging images. We compared PVS burden between PD and HC, and conducted partial correlation analysis between PVS burden and clinical and imaging features. PD patients had a significantly more serious PVS burden in BG and midbrain, and the PVS number in BG was significantly correlated to the PD disease severity and L-dopa equivalent dosage. The fractional anisotropy and mean diffusivity values of certain subcortical nuclei and white matter fibers within or nearby the BG and midbrain were significantly correlated with the ipsilateral PVS burden indexes. Regarding to the midbrain, the difference between bilateral PVS burden was, respectively, correlated to the difference between fiber counts of white fiber tract passing through bilateral substantia nigra in PD. Our study suggests that PVS burden indexes in BG are candidate biomarkers to evaluate PD motor symptom severity and aid in predicting medication dosage. And our findings also highlight the potential correlations between PVS burden and both grey and white matter microstructures.

## Introduction

Perivascular spaces (PVSs) are fluid-filled spaces surrounding blood vessels that course from the brain surface through brain parenchyma^1^. It’s a normal anatomical structure in the central nervous system, commonly observed in basal ganglia (BG), white matter centrum semiovale, midbrain and hippocampus^2^. Although the results of PVS studies remained controversial, the PVS burden had been shown to correlate with aging, cognitive capacity^3,4^, sleep quality^5^, and depressive states^6^. It was described as a characteristic of neurological diseases including, small vessel disease^7^, Alzheimer’s disease (AD)^8^, multiple sclerosis^9^, as well as Parkinson’s disease (PD)^10,11^.

PD is a chronic neurodegenerative disorder, characterized by motor symptoms (resting tremor, bradykinesia, rigidity, and gait abnormalities) and non-motor symptoms (depression, anxiety, cognition decline, etc.)^12^. The pathological hallmarks of PD are dopaminergic neuron degeneration and abnormal α‐synuclein deposition in the substantia nigra (SN)^13^. Recent studies indicated that the PVS system played a disease-modifying role in PD^10,11,14,15^. Since the PVS system is involved in the glymphatic drainage system to eliminate metabolic waste^16,17^, and there was evidence showing that PVS system helped drive out soluble proteins involved in neurodegenerative diseases from the brain interstitial fluid^18^, we also assume that PVS participates in the clearance of abnormal α‐synuclein^19^ and its dysfunction may aggravate the pathology of PD.

Enlarged PVS became visible and measurable through magnetic resonance imaging (MRI) techniques. PVS burden was defined as indexes of PVS quantification to represent severity of PVS enlargement, including PVS number and volume^20^. Previous studies reported that PVS might contribute to the characteristic asymmetry of motor symptoms^10^, and could be a useful neuroimaging biomarker for evaluating cognitive decline in PD^10,15^. The physiological and pathophysiological mechanisms of PVS may be reflected in its neighboring white matter, as PVS burden had shown correlation with white matter hyperintensity^21^.

These studies partly explained the significance of PVS, however, it was previously subject to the limitation of MRI resolution. Only PVSs with larger diameters were visible, and most studies focused primarily on the potential pathogenicity of enlarged PVSs. Due to the higher resolution and signal-to-noise ratio (SNR) of 7 Tesla (7 T) MRI, more small size PVSs can be detected and the PVS numbers were markedly higher than conventional 1.5 or 3 T MRI studies^1^. Therefore, the small PVS should also be taken into account in studies of neurodegenerative diseases, which could alter the correlations between PVS burden and clinical features compared to previous studies^1^.

This study aimed to compare the PVS burden in early-stage PD to those in healthy controls (HCs), with the influence of smaller PVSs included. We performed a quantitative evaluation of PVS burden on 7 T MRI images to explore the prevalence of PVS and characterize the correlation patterns of PVS burden with clinical and imaging features to further understand its pathophysiological mechanism in PD.

## Results

### Participant characteristics

The demographic and clinical characteristics of the PD and HC participants are shown in Table 1. There were no significant differences between the PD and HC groups in age, sex, education, Hamilton Rating Scale for Anxiety (HAMA) score, Mini-Mental State Examination (MMSE) score, Montreal Cognitive Assessment (MoCA) score, Parkinson Neuropsychometric Dementia Assessment (PANDA) score, or prevalence of vascular risk factors such as hypertension, hyperlipidemia, diabetes mellitus, and smoking. The PD patients had significantly higher Hamilton Rating Scale for Depression (HAMD) score than the HC participants (*p* = 0.009), indicating the depressive state was more severe in the PD patients.

**Table 1:** Demographic and clinical characteristics of participants.

	PD (*n* = 40)	HC (*n* = 41)	*p*
Age (years)	52.5 ± 1.2	49.8 ± 2.0	0.242
Sex, male (%)	47.5	51.2	0.738
Education (years)	8.8 ± 0.7	10.8 ± 0.7	0.059
Hypertension (%)	25.0	19.5	0.553
Diabetes mellitus (%)	5.0	4.9	0.626
Hyperlipidemia (%)	0.0	4.9	0.485
Smoking (%)	17.5	19.5	0.816
Disease duration (years)	5.4 ± 1.0	–	–
MDS-UPDRS I	5.5 ± 0.5	–	–
MDS-UPDRS II	8.4 ± 0.6	–	–
MDS-UPDRS III	23.4 ± 2.0	–	–
MDS-UPDRS IV	1.9 ± 0.5	–	–
MDS-UPDRS total	39.1 ± 3.0	–	–
H-Y stage	1.8 ± 0.1	–	–
LEDD (mg)	535.0 ± 39.5	–	–
HAMA	3.7 ± 0.5	2.7 ± 0.5	0.083
HAMD	5.8 ± 0.6	3.8 ± 0.6	0.009**
MMSE	26.5 ± 0.5	27.3 ± 0.4	0.272
MoCA	22.6 ± 0.8	23.8 ± 0.7	0.285
PANDA	20.7 ± 0.9	21.3 ± 1.1	0.672

Results are expressed as means ± standard error of the mean for the continuous variables and as frequencies for the categorical variables.

*PD* Parkinson’s disease, *HC* healthy control, *MDS-UPDRS* Movement Disorder Society-sponsored revision of the Unified Parkinson’s Disease Rating Scale, H-Y stage Hoehn & Yahr stage, *LEDD* L-dopa equivalent daily dose, *HAMA* Hamilton Rating Scale for Anxiety, *HAMD* Hamilton Rating Scale for Depression, *MMSE* Mini-Mental State Examination, *MoCA* Montreal Cognitive Assessment, *PANDA* Parkinson neuropsychometric dementia assessment.

**Indicates *p* < 0.01.

### Distribution of PVSs

**Fig. 1:**
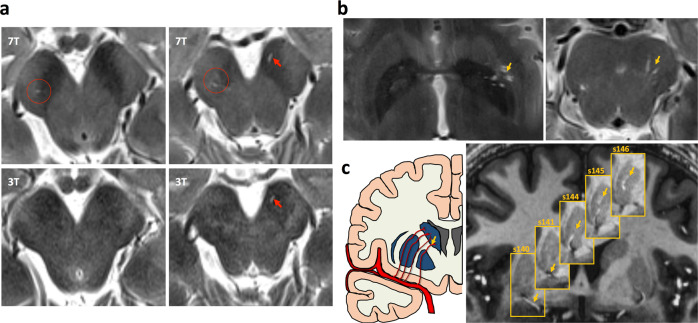
Visualization of the perivascular space. **a** Comparison of detectable PVS in one subject on 7 T images and 3 T images. **b** PVSs in the BG and midbrain on T2 images. **c** Visualization of the PVS system and its spatially corresponding lenticulostriate artery in the BG on MP2RAGE imaging.

Although our 3 T MRI sequence already achieved a relatively high resolution, 7 T MRI images could expose PVSs more clearly, particularly for small size PVSs due to its much higher SNR compared to 3 T MRI images (Fig. 1a). The PVS quantification was performed by a single reader (T.S.) blind to the participants’ clinical status. Before the formal study, reader (T.S.) was trained by a senior researcher (Y.Y.) on a training set of 20 cases^21^. Intra-class correlation coefficients between two readers reached 0.81 for PVSs in BG and 0.89 for PVSs in midbrain.

**Fig. 2:**
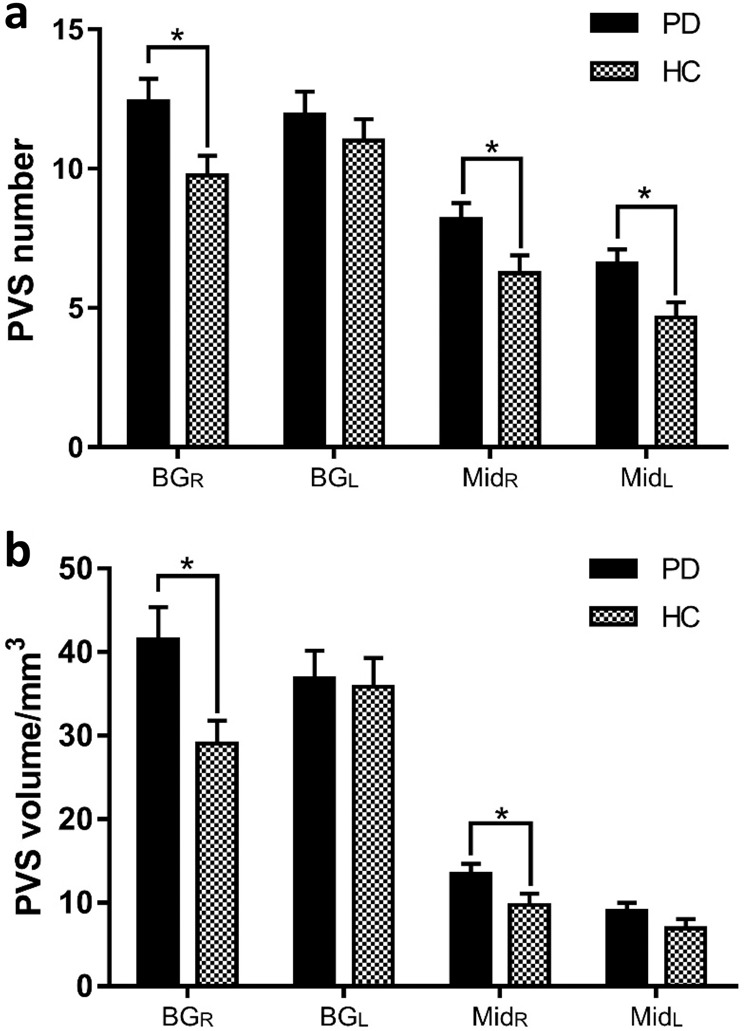
Comparison of the perivascular space burden between PD and HC groups. **a** PVS number and **b** PVS volume in BG and midbrain (Mid). *Indicates *p* < 0.05. The results are presented as means ± standard error of mean.

In this study, the PVS burden indexes, PVS number and PVS volume, in the right-hemispheric BG (BG_R_), left-hemispheric BG (BG_L_), right-hemispheric midbrain (Mid_R_), and left-hemispheric midbrain (Mid_L_) were shown in Table 2. Both PVS number and volume in BG_R_ were significantly larger in the PD group than those in the HC group (*p* = 0.035 for number, *p* = 0.015 for volume). And the PVS number in bilateral midbrain (*p* = 0.042 for Mid_R_, *p* = 0.018 for Mid_L_) and the PVS volume in Mid_R_ (*p* = 0.041) were significantly larger in the PD group compared to the HC group, as shown in Fig. 2.

**Table 2:** PVS distribution in PD and HC.

	PD (*n* = 40)	HC (*n* = 41)	*p*
*BG*_*R*_			
Number	12.4 ± 0.8	9.81 ± 0.7	0.035*
Volume	41.5 ± 3.9	29.1 ± 2.7	0.015*
*BG*_*L*_			
Number	11.9 ± 0.8	11.1 ± 0.8	0.555
Volume	36.9 ± 3.3	35.6 ± 3.4	0.921
*Mid*_*R*_			
Number	8.2 ± 0.6	6.2 ± 0.6	0.042*
Volume	13.5 ± 1.2	9.6 ± 1.4	0.041*
*Mid*_*L*_			
Number	6.6 ± 0.5	4.6 ± 0.5	0.018*
Volume	9.1 ± 1.0	6.8 ± 1.1	0.145

Results are expressed as means ± standard error of the mean.

*PVS* perivascular space, *PD* Parkinson’s disease, *HC* healthy control, *BG* basal ganglia, *Mid* midbrain, *R* right-hemisphere, *L* left-hemisphere.

*Indicates *p* < 0.05.

### Correlation analysis with clinical features

**Fig. 3:**
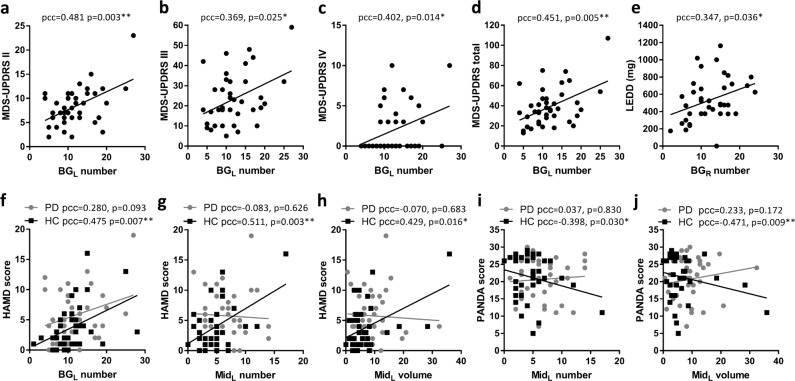
Correlations of perivascular space burden indexes in BG and midbrain with clinical features. Correlations with **a**−**d** MDS-UPDRS scores and **e** LEDD in PD. Correlations with **f**−**h** HAMD score in PD and HC. Correlation with **i**−**j** PANDA score in PD and HC. *Indicates the partial correlation coefficient (pcc) < 0.05 and ** indicates pcc < 0.01.

After regressing out the effect of age, sex, and total brain volume, the PVS number of BG_L_ was positively correlated with Movement Disorder Society-sponsored revision of the Unified Parkinson’s Disease Rating Scale (MDS-UPDRS) II subscore (partial correlation coefficients (pcc) = 0.481, *p* = 0.003), III subscore (pcc = 0.369, *p* = 0.025), IV subscore (pcc = 0.402, *p* = 0.014), and total score (pcc = 0.451, *p* = 0.005) in the PD group (Fig. 3a–d), while the PVS volume of BG_L_ was not correlated to MDS-UPDRS scores. The PVS number of BG_R_ was positively correlated to L-dopa equivalent daily dose (LEDD) (pcc = 0.347, *p* = 0.036, Fig. 3e). However, the PVS burden indexes were not correlated to HAMA, HAMD, MMSE, MoCA, or PANDA scores.

In the HC group, the PVS number of BG_L_ (pcc = 0.475, *p* = 0.007), the PVS number of Mid_L_ (pcc = 0.511, *p* = 0.003) and the PVS volume of Mid_L_ (pcc = 0.429, *p* = 0.016) were positively correlated with the HAMD score after regressing out the effect of age, sex, and total brain volume (Fig. 3f–h). Furthermore, the PVS burden indexes in Mid_L_ were negatively correlated to the PANDA score (pcc = −0.398, *p* = 0.030 for PVS number; pcc = −0.471, *p* = 0.009 for PVS volume), as shown in Fig. 3i, j.

### Correlation analysis with FA and MD values

**Fig. 4:**
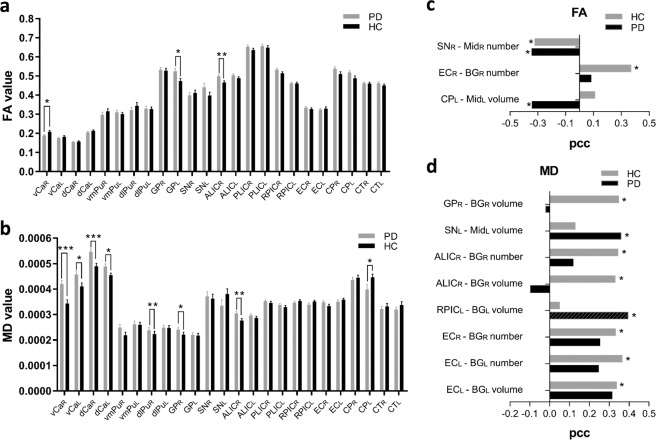
Fractional anisotropy (FA) values and mean diffusivity (MD) values analysis. **a** and **b** Comparison of FA and MD values between PD and HC groups. **c** and **d** Correlations between PVS burden indexes and FA, MD values in PD and HC. * Indicates *p* < 0.05, ** indicates *p* < 0.01, *** indicates *p* < 0.001. The results are presented as means ± standard error of mean. *Abbreviations*: vCa ventral caudate, dCa dorsal caudate, vmPu ventromedial putamen, dlPu dorsolateral putamen, GP globus pallidus, SN substantia nigra, ALIC anterior limb of internal capsule, PLIC posterior limb of internal capsule, RPIC retrolenticular part of internal capsule, EC external capsule, CP cerebral peduncle, CT corticospinal tract, pcc partial correlation coefficient.

Results of the regions of interest (ROI)-based analysis showed that the PD group had significantly lower fractional anisotropy (FA) value in right-hemispheric ventral caudate (vCa) (*p* = 0.031) and higher FA value in left-hemispheric globus pallidus (GP) (*p* = 0.031), as well as higher mean diffusivity (MD) values in bilateral vCa (*p* = 0.0003 for right-hemisphere, *p* = 0.030 for left-hemisphere), bilateral dorsal caudate (dCa) (*p* = 0.0006 for right-hemisphere, *p* = 0.021 for left-hemisphere), right-hemispheric ventromedial putamen (vmPu) (*p* = 0.027), and right-hemispheric GP (*p* = 0.049). In the fiber of interest (FOI)-based analysis, we also found significantly higher FA value in right-hemispheric anterior limb of internal capsule (ALIC) (*p* = 0.005), as well as higher MD value in right-hemispheric ALIC (*p* = 0.005) and lower MD values in left-hemispheric cerebral peduncle (CP) (*p* = 0.029) in PD group compared to HC group (Fig. 4a, b). The results of right vCa and right dCa were survived after false discovery rate (FDR) correction.

Correlations between the FA values and the PVS burden indexes were presented in Fig. 4c. The FA value of subcortical nuclei, such as the right-hemispheric SN within midbrain was negatively correlated with the PVS number for the Mid_R_ (pcc = −0.342, *p* = 0.044) in PD group, whereas a similar presentation was also found in HC group (pcc = −0.322, *p* = 0.049). Regarding to the white matter fibers, the FA value of the left-hemispheric CP was negatively correlated with the PVS volume of Mid_L_ (pcc = −0.341, *p* = 0.045) in the PD group. And in the HC group, the PVS number of BG_R_ was positively correlated with FA values of right-hemispheric external capsule (EC) (pcc = 0.369, *p* = 0.023).

Correlations between MD values and PVS burden indexes were presented in Fig. 4d. The MD value of left-hemispheric SN was positively correlated with the PVS volume of Mid_L_ (pcc = 0.359, *p* = 0.034) in the PD group. In the HC group, the MD value of right-hemispheric GP was positively correlated with the PVS volume of BG_R_ (pcc = 0.346, *p* = 0.033). In regard to the white matter fibers, the PVS volume of BG_L_ was positively correlated with the MD value of left-hemispheric retrolenticular part of internal capsule (RPIC) (pcc = 0.393, *p* = 0.019) in the PD group. In the HC group, the MD value of right-hemispheric ALIC was positively correlated with the PVS number (pcc = 0.342, *p* = 0.035) and volume (pcc = 0.330, *p* = 0.043) of BG_R_. The MD value of right-hemispheric EC was positively correlated to the PVS number of BG_R_ (pcc = 0.330, *p* = 0.043), and the MD value of left-hemispheric EC was positively correlated to the PVS number (pcc = 0.363, *p* = 0.025) and volume (pcc = 0.337, *p* = 0.039) of BG_L_.

### Correlation analysis with white matter fibers

**Fig. 5:**
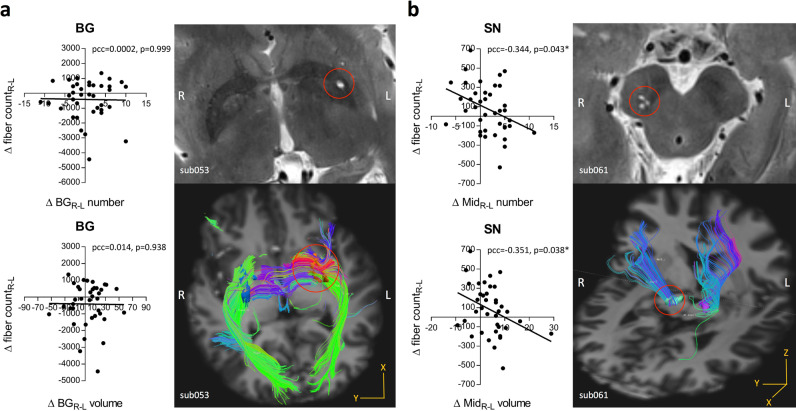
White matter fibers tacking analysis. **a** Correlations of the difference between bilateral PVS burden indexes in BG (ΔBG_R-L_ number and volume) and the difference between fiber counts of white fiber tract passing through the bilateral BG in PD. An example of fiber tacking result of one PD patient (sub053) with severer PVS burden in left-hemispheric putamen. **b** Correlations of the difference between bilateral PVS burden indexes in midbrain (ΔMid_R-L_ number and volume) and the difference between fiber counts of white fiber tract passing through the bilateral midbrain in PD. An example of fiber tacking result of one PD patient (sub061) showing severe PVS burden and sparser fiber in right midbrain. * Indicates the partial correlation coefficient (pcc) with *p* < 0.05. Δfiber count_R-L_ difference between fiber counts of white fiber tract passing through the bilateral BG or midbrain.

There was no significant difference of the fiber counts of white fiber tract passing through the nuclei within BG and midbrain between PD and HC groups, and we did not find significant correlations between PVS burden and fiber counts. Regarding to the BG region, the difference between bilateral PVS burden indexes (both PVS number and volume) were not significantly correlated to the difference between fiber counts of white matter fiber tract passing through bilateral subcortical nuclei, including the vCa, dCa, vmPu, dorsolateral putamen (dlPu), GP, and whole BG in both PD and HC groups (only results of whole BG in PD was shown in Fig. 5a). And with respect to the midbrain region, the difference between bilateral PVS burden indexes were, respectively, correlated to the difference between fiber counts of white fiber tract passing through bilateral SN (pcc = −0.344, *p* = 0.043 for PVS number; pcc = −0.351, *p* = 0.038 for PVS volume) after controlling for total brain volume, age, and sex in the PD group (Fig. 5b). Although there was no statistical significance, the HC group still showed a negative correlation trend.

## Discussion

In this study, we explored the PVS burden considering both number and volume in BG and midbrain in the early-stage PD patients. We detected significantly heavier PVS burden in PD group than the HC group on 7 T MRI images, as well as provided fresh data to support the relationship between PVS burden and motor symptom severity in PD. Along with previous studies, we also found that PVS burden was significantly correlated with cognitive function and emotional status. Furthermore, changes of diffusion tensor imaging (DTI)-derived features of certain subcortical nuclei and white matter fibers were also associated with PVS burden.

Considering the potential functions of the PVS system, studies had proved that the PVS waste drainage system contributed to a larger portion of interstitial solutes clearance, such as amyloid beta (Aβ), preventing Aβ deposition that was thought to be pathogenic in AD^18,22^. Similar to AD, abnormal aggregated protein α-synuclein that forms Lewy bodies or Lewy neuritis is the main neuropathological characteristics of PD^13^. PVS may also contribute to clearance of abnormal α-synuclein in the brain^19^. On the one hand, the failure of perivascular clearance might cause increased α-synuclein deposition, formation of Lewy pathology and damage of dopaminergic neurons, which might lead to modification of PD pathology and clinical features. On the other hand, dopaminergic neuronal degeneration might produce excessive metabolic wastes and accumulated in PVS, lead to perivascular blockage and heavier PVS burden, which might contribute to the pathological process in PD^23^. Unfortunately, previous studies linking PVS and dopamine transporter (DAT) uptake showed inconsistent results. Several case reports and case-control studies had shown that the existence of PVS might relate to lower DAT uptake^24–26^. And it was also proposed that enlarged PVSs would cause downstream dysfunction from the presynaptic nigrostriatal dopaminergic system and led to incomplete response to levodopa^25,26^. Other study showed no significant correlation between PVS burden and DAT positron emission tomography (PET) abnormality^11^. To explain this phenomena, studies had identified two types of PVS according to the histological appearance of the surrounding tissue: type 1 with normal surrounding nervous tissue, and type 2 with surrounding rarefication and abnormal gliosis^11,27^, suggesting that these inconsistent results might be due to different types of detected PVS in these studies. The most commonly detected PVSs on MRI images have diameters <3 mm, are used to be considered as type 1 with normal physiological functions. Accordingly, the present study mainly focused on the potential role of these PVSs.

Previous studies mainly focused on reporting PD cases with enlarged PVS^14,25,28,29^ and studying the populations of PD patients without comparing to the normal controls^15,30,31^. We compared the PVS burden of PD group to those of HCs, and found a more severe PVS burden in PD. Another difference with previous studies was that those studies mainly focused on middle-stage and late-stage PD patients with average of ages over 60 years old^10,11^, whereas we intended to investigate the PVS burden in early-stage PD patients with average of age about 50 years old. We observed more severe PVS burden in the PD group, therefore we revealed that the exacerbation of PVS burden was already existed in the early phase of PD disease process. Statistically significant differences were mainly found in PVS burden in the right-hemisphere, the left hemisphere only showed an increasing trend in PD. To a certain extent, our results might provide new evidence for hemispheric lateralization among PD. The progress of PVS burden in PD might start from right-hemisphere, and irrelevant to symptom onset-side. Previous studies have pointed out the lateralization of certain brain function, the right-hemisphere is dominant for controlling limb position and posture, whereas the left-hemisphere is the dominant for appendicular movements^32,33^. The neurodegenerative process of PD also initials asymmetrically, might exhibit left-hemispheric predominance of subclinical nigrostriatal deficit^34,35^. Moreover, PD patients with freezing of gait were presented with more affected right-hemisphere circuitry, especially in the pedunculopontine nucleus within midbrain^36,37^. However, the mechanism of lateralization for motor features and neuro-pathophysiology of PD still requires more evidence. In the present study, significant correlations between the PVS burden in left BG and MDS-UPDRS scores were also found in the PD patients. We suggested that the PVS number in left BG could reflect severity and progression of PD motor symptoms. In addition, our results showed that PD patients with more severe PVS burden in right hemisphere needed higher dosage of anti-PD drugs, which is in line with the hypothesis that possible dysfunction of the PVS pathway existed in PD, as well as the incomplete response to levodopa caused by PVS-related dysfunction of presynaptic nigrostriatal dopaminergic system^25,26^. The right striatum might have lower quantities of dopamine than those of left, which lead to vulnerability to nigrostriatal denervation in right-hemisphere^38^. Interestingly, PD patients with relatively heavier right-hemisphere dysfunction displayed greater medication doses^39^, which was consistent with our results. Besides, since only the PVS number of BG was correlated to these clinical features, the PVS number rather than PVS volume in BG might be a more important measure to represent severity of motor symptoms, which was consistent with one previous study declaring that PVS number was more meaningful than PVS volume^21^.

A previous study reviewed that the prefrontal-limbic network in depression is modulated by hypothalamus, BG, and midbrain^40^. Increased PVS burden over time significantly associated with higher incidence of depressive symptoms^6^ and might be able to predict resistance to antidepressant monotherapy in elderly patients with depression^41^. An animal study supported this assumption with revealing that dilated PVS might exacerbate depression-like behaviors by increasing the levels of inflammatory factors^42^. We found significant association between HAMD score and PVS burden of both BG and midbrain in the HC group, indicating that poor emotional states like depression might aggravate the PVS burden. But the emotional status of most HCs was at a normal level, the correlation might be in different extent while focusing on patients with depression. As PVS dysfunction might relate to neuro-neurophysiology and clinical motor features of PD, it also related to cognitive decline in PD^3,4,15^. The PVS burden, particularly correlated with executive functioning^4^ and visuospatial ability^43^, which were frequently involved cognitive domains in PD. Possible mechanism of PVS might be decreased clearance of metabolic waste, lead to accumulation of Aβ, α-synuclein relating to cognitive decline. In patients with cognition decline, brain atrophy was often accompanied by cerebrospinal fluid (CSF) space enlargement and cerebral ventricular dilatation, might following by a secondary ex-vacuo effect as compensation^3,44^. Therefore, the PVS might be an earlier imaging feature than brain atrophy during the aging and cognition decline process^3^. Studies of PD provided evidence that PVS burden in BG could be regarded as biomarker to evaluate cognitive decline in PD^15,30^. We also found that healthy elderly with more severe PVS burden in midbrain, had more decreased cognitive function. However, the PVS burden did not show a significant correlation with the emotional state and cognitive function in PD patients, which might be caused by the possible presence of other influential factors that covered these correlations.

PVS is a microscopic tubular structure that occupies extra-vascular space, in which water molecules move freely^45^. Regarding to white matter fiber tracts, they are spatially parallelizing with PVSs^46^. Except for vessels, water molecules inside the PVS are also hindered by surrounding brain tissue. DTI-derived features reflect the diffusion properties of both the surrounding brain tissue and fluid from PVS, thus the enlargement of PVS might have substantial influence on DTI features^45^. At the same time, the activity of the glymphatic system could be evaluated by calculating diffusivity along the PVS, providing more evidence that the PVS burden related to the impairment of white matter fiber^47^. In addition to the relationship between PVS and dopaminergic terminals that we have mentioned above^11^, gray and white matter microstructures might also be an important part of the pathophysiological mechanism of PVS.

We used the FA value to quantify the degree of directional dependence of diffusion that depends on the number and density of axons^48^. The MD value was also used to describe the overall amount of isotropic diffusion within brain tissue^49^, and its increasing might be caused by enlargement of extracellular space, suggesting degeneration of brain tissue^50^. However, towards the changes of white matter microstructure in PD, the alterations of FA and MD due to PD remain controversial. Most studies focusing on advanced-stage PD patients reported decreased FA and/or increased MD, indicating demyelination and axonal degeneration of white matter microstructure^51^. Other results like increased FA and decreased MD were defined as microstructural compensation in prodromal-stage and early-stage of PD, which was distinguished from pathological findings^52^. Our results showed significantly decreased FA in caudate accompanied by increased MD in caudate and putamen, demonstrating degeneration in these respective regions. And significantly increased FA and MD values in GP and ALIC were detected, indicating possible selective neurodegeneration or a potential compensatory reorganization after treatment^50^.

Back to the correlation between PVS burden and surrounding brain tissues, the PVS number in BG was correlated with the load of deep white matter hyperintensity, which was corresponding to the demyelination and sparseness of axons^21^. And some PD cases reported that the DTI features of fibers adjacent to enlarged PVS were changed compared to the normal contralateral fibers^28^. Similarly, our results indicated that PVS burden severity was related to the integrity of adjacent white matter fibers. The participants with higher PVS volume in midbrain had a more decreased FA and more increased MD in SN, revealed that the PVS might increase the diffusivity of water molecules and even exacerbate the degeneration of SN. The MD values of GP, ALIC, RPIC, and EC were also increasing with the PVS burden indexes of BG, indicating PVS burden in BG also influenced the adjacent white matter microstructure. We also investigated the correlation between the fiber counts of white matter fiber track passing through bilateral subcortical nuclei and bilateral PVS burden. For the midbrain in PD patients, we found that the hemisphere with more severe PVS burden in midbrain showed a sparser fiber track passing through SN than the contralateral hemisphere. Since the HCs had less severe PVS burden than PD patients, they just showed a negative correlation trend, with no statistical significance was attained. PVS burden in unilateral cerebral hemisphere was significantly correlated to ipsilateral DTI-derived features, while the same index in contralateral hemisphere only showed a correlation trend. Possibly, we quantitated the PVS burden in the whole BG and midbrain, within which the distribution of PVS might vary in different subcortical nuclei and white matter fibers. Although without statistical significance, these results still indicating slightly positive or negative correlations between PVS burden and both gray and white matter microstructure. Based on these results, smaller PVS burden that was previously considered as type 1 “normal” structure might still exhibit physiological dysfunctions, which was mainly reflected by causing an indispensable important effect on the brain parenchyma, particularly the DTI-derived features. However, this phenomenon might not be unique to PD. As a possible biomarker for cerebral small-vessel disease affecting nearby brain parenchyma^21^, PVS burden indexes might be able to play a disease-modifying role in a certain disease by involving related brain regions.

Growing evidence had shown that insufficient clearance of pathologic protein deposits through brain drainage system including the PVS pathway might serve as new targets for promising modifier treatment and even prevention for neurodegenerative disorders^53^. The “perivascular pump” driven by arterial pulsation might be a powerful convection enhanced delivery method, by which viral particles for gene-therapy or therapeutic agents could be administered directly into central nervous system^54^. Furthermore, focused ultrasound combined with microbubbles could enhance the brain blood barrier opening along with expansion and contraction of PVS, which might induce bulk flow and lead to increased penetration of drugs into the brain tissue^55^. Another important therapeutic target is enhancing the brain clearance mechanisms by reducing in reactive astro- and microgliosis, normalizing the perivascular aquaporin-4 polarization, inducing fibrinolysis of fibrin clots in PVS, which are still beyond clinical application so far^56^. Since the clearance of interstitial solutes increases by 60% during the sleep state and exercise could also increase glymphatic influx and clearance, sleep improvement, and voluntary exercise provide easily implemented strategies to improve glymphatic function^56^. Moreover, increased PVS burden is associated with hypertension^56^, thus, improved hypertension control might also help in slowing down the aggravation of PVS burden and its potential role in neurodegenerative disorders.

In conclusion, the present study demonstrated that the PVS burden in the BG and the midbrain were already aggravated in early-stage PD patients. The PVS burden indexes, including PVS number and volume, could be considered as a biomarker to evaluate severity and progression of PD motor symptom, and aid in predicting medication dosage. Furthermore, the PVS burden might also reflect microstructural alteration of surrounding brain tissues. Despite the limited sample size, with higher spatial resolution and SNR of MRI data, our study provides preliminary evidence to support the potential role of PVS in the pathophysiology of PD. Future work will focus on reviewing the correlations between PVS burden and the progression of clinical feature, as well as the continuing impact of PVS burden on brain parenchyma through longitudinal research during the course of PD.

## Methods

### Participants and clinical data collection

This study was approved by the ethics committee of Second Affiliated Hospital of Zhejiang University School of Medicine. Written informed consent was obtained from all the participants. We recruited 40 PD patients from the outpatient clinic of the Department of Neurology of Second Affiliated Hospital of Zhejiang University School of Medicine, and 41 age-matched and sex-matched healthy controls (HCs) from neighboring communities.

PD patients met the Movement Disorder Society (MDS) Clinical Diagnostic Criteria for PD^57^. HCs had no history of neurological or psychiatric disease and no family history of PD or related neurodegenerative disorders. Participants with small vessel disease such as white matter hyperintensity, lacunar infarctions, and cerebral microbleeds were excluded. All the participants underwent a clinical evaluation, including clinical histories (self-report or medical records), neurological, and neuropsychological examinations. PD disease severity was assessed using MDS-UPDRS and Hoehn & Yahr stage (H-Y stage) during off-stage, early-stage PD was defined as H-Y stage between 1 and 2.5^58^. We separately analyzed the MDS-UPDRS subscores for more detailed information, including the subscore I for non-motor experiences of daily living, subscore II for motor experiences of daily living, subscore III for motor examination, and subscore IV for motor complications^59^. We applied the HAMA and HAMD to assess emotional status, the MMSE, MoCA, and PANDA to evaluate cognitive function.

### MRI images acquisition

MRI images were acquired with a 7 T Magnatom research system (Siemens Healthcare, Erlangen, Germany) with prototype sequences, including a magnetization prepared with two rapid gradient echoes (MP2RAGE) sequence (voxel size: 0.7 × 0.7 × 0.7 mm^3^, TR = 5000 ms, TI1/TI2 = 900/2750 ms, TE = 2.3 ms, *α*1/*α*2 = 5°/3°, and two times generalized autocalibrating partial parallel acquisition (GRAPPA) acceleration), a T2-weighted turbo spin echo (TSE) sequence (voxel size: 0.5 × 0.5 × 2.4 mm^3^, TR = 7000 ms, and TE = 66 ms) and a multiband echo-planar DTI sequence (voxel size: 1.5 × 1.5 × 1.5 mm, TR = 6000 ms, TE = 87.4 ms). A patient underwent an extra 3 T MRI scan to obtain another T2 image on a 3 T Prisma research system (Siemens Healthcare, Erlangen, Germany) using a T2-weighted TSE sequence (voxel size: 0.5 × 0.5 × 2.8 mm^3^, TR = 9360 ms, and TE = 100 ms) for comparsion.

### MRI images processing

In this study, we focused on the effects of small size PVSs. Therefore, a PVS was defined as a small (<3 mm), smooth, linear-shaped, ovoid-shaped, or round-shaped space with isointense CSF that ran along with perforating arteries^60–62^. We acquired a high-resolution MP2RAGE image to visualize the PVS system and its spatially corresponding lenticulostriate artery (LAS) simultaneously, as shown in Fig. 1c, with PVSs showing low intensity similar to CSF and the blood vessels showing high intensity. The PVS burden was defined to represent the severity of PVSs, which was assessed on axial T2-weighted images in the BG and midbrain (Fig. 1b). According to previous studies^15,63^, we performed PVS quantification in the slice containing the maximum amount of PVSs in the BG region^60^, of which the anterior and posterior border zones were the anterior end of the insula and the posterior end of the thalamus^1^. For the midbrain region, the PVSs were counted in the whole volume^64^. The PVS counting, segmentation, and volume calculation were performed using the ITK-SNAP software version 3.8 (http://www.itksnap.org/). We separately counted PVS number and calculated the PVS volume in four defined regions: BG_R_, BG_L_, Mid_R_, and Mid_L_. We manually delineated along the boundary of all identified PVSs. The ITK-SNAP software automatically gave the voxel number of identified PVSs in each region, and the PVS volume was calculated as the sum of the individual volume of identified PVS in each region per mm^3^. Then we obtained the PVS number and the PVS volume of each region, regarded as the PVS burden indexes.

Total brain volume that was expressed as the sum of total gray and white matter volume was estimated on MP2RAGE images using VBM8 embedded in SPM8 software (Statistical Parametric Mapping; https://www.fil.ion.ucl.ac.uk/spm/software/spm8/). The DTI data was processed using FSL version 6.0.1 (FMRIB Software Library; http://www.fmrib.ox.ac.uk/fsl) to generate fractional anisotropy (FA) and mean diffusivity (MD) maps. The Brainnetome Atlas^65^ adding additional nuclei within midbrain, and the ICBM-DTI-81 white matter atlas^66^ were separately transformed into each participant’s DTI space^67^. Then we used “fslmeants” command of FSL to obtain 12 subcortical nuclei ROIs, including the bilateral vCa, dCa, vmPu, dlPu, GP and SN, as well as 12 FOIs, including the bilateral ALIC, posterior limb of internal capsule (PLIC), RPIC, EC, CP, and corticospinal tract (CT) from these two atlases. Then the averages of FA and MD values were extracted for subsequent analysis.

Deterministic tractography was conducted using Diffusion Toolkit version 0.6.4 and TrackVis version 0.6.1 (http://trackvis.org) in each participant’s DTI space. We applied a DWI mask threshold and an angular threshold of 35° to limit deviations from main path. We loaded ROIs from transformed atlas including bilateral vCa, dCa, vmPu, dlPu, GP, and SN to track white matter fibers passing through these subcortical nuclei, and the fiber counts of each track were given for subsequent analysis.

### Statistical analysis

The results were expressed as mean ± standard error of mean (SEM) for continuous variables and as percentages for the categorical variables. The Student’s two-sample *t*-test was used for the comparison of continuous variables including clinical and DTI features between groups. The chi-squared test was used for the comparison of categorical variables. The difference between right- and left-hemispheric PVS burden indexes, and the differences between fiber counts of white matter fibers passing through the right- and left-hemispheric nuclei within BG and midbrain were also calculated. Partial correlation analysis was performed to calculate pcc and evaluate the correlations between PVS number, PVS volume and MDS-UPDRS, H-Y stage, LEDD, MMSE, MoCA, PANDA, HAMA, HAMD scores, and DTI parameters. Age, sex, education, and total brain volume were entered as covariates due to their potential influence. These analyses were considered significant at *p* < 0.05, uncorrected. At the same time, we further used a FDR corrected *p* < 0.05 for multiple testing.

Statistical analyses were conducted with SPSS Statistics software version 22 (IBM Corporation, Armonk, NY, USA). Statistical plots were generated by GraphPad Prism version 7 (GraphPad Inc., San Diego, CA, USA).

### Reporting summary

Further information on research design is available in the Nature Research Reporting Summary linked to this article.

## Supplementary information

Reporting Summary

## Data Availability

Clinical and neuroimaging data can be shared on reasonable request from qualified investigators by contacting the corresponding authors. Sharing and reuse of our data require the expressed written permission of the authors.

## Code availability

As previously mentioned in the “MRI images processing” subsection, the PVS quantification was performed using the ITK-SNAP version 3.8 (http://www.itksnap.org/). Total brain volume was estimated using VBM8 embedded in SPM8 software (Statistical Parametric Mapping; https://www.fil.ion.ucl.ac.uk/spm/software/spm8/). The DTI data was processed using FSL 6.0.1 (FMRIB Software Library; http://www.fmrib.ox.ac.uk/fsl). Deterministic tractography was conducted using Diffusion Toolkit 0.6.4 and TrackVis 0.6.1 (http://trackvis.org). Statistical analyses were conducted with SPSS Statistics software version 22 (IBM Corporation, Armonk, NY, USA). Statistical plots were generated by GraphPad Prism version 7 (GraphPad Inc., San Diego, CA, USA).
